# Assessing change and establishing empirical cutoffs: the Brief INSPIRE-O measure for personal recovery in mental health services

**DOI:** 10.1007/s00127-025-02948-7

**Published:** 2025-06-24

**Authors:** Stine Bjerrum Moeller, Pia Veldt Larsen, Stephen F. Austin, Mike Slade, Ida-Marie T. P. Arendt, Lotte Kring, Sebastian Simonsen

**Affiliations:** 1 Psychotherapy Research Unit, Mental Health Centre Stolpegaard, Capital Region Psychiatry, Gentofte, Denmark; 2 Department of Psychology, University of Southern Denmark, Danish National Center of Psychotraumatology, Campusvej 55, Odense M, DK-5230 Denmark; 3 Mental Helath services in the Region of Southern Denmark, Department of Multidisciplinary Traumatreatment, Vejle, Denmark; 4 Mental Health Services East, Psychiatric Research Unit, Psychiatry Region Zealand, Roskilde, Denmark; 5 Department of Psychology, University of Copenhagen, Copenhagen, Denmark; 6 School of Health Sciences, Institute of Mental Health, University of Nottingham, Nottingham, UK; 7 Health and Community Participation Division, Nord University, Bodø, Norway

**Keywords:** Mental health, INSPIRE, Personal recovery, Transdiagnostic, Sensitivity to change, Empirical cutoff, Clinical improvement, ROC

## Abstract

**Introduction:**

Personal recovery in mental health services, encouraged by the World Health Organization, has gained significance in research and clinical settings. However, measuring personal recovery remains challenging due to the lack of universally accepted instruments. This study assessed Brief INSPIRE-O’s ability to map personal recovery by determining cut-off scores and its ability to detect change in the process of personal recovery.

**Method:**

Data was from the internet-based monitoring system (IMS) at the Mental Health Service, Capital Region of Denmark. Between 2018 and 2020, 8,192 patients with baseline data on Brief INSPIRE-O were included to assess its role in measuring personal recovery. Additionally, for analyses focusing on Brief-INSPIRE-O as a measure of change in personal recovery, we included 2,714 patients with pre- and post-treatment data.

**Materials:**

Brief INSPIRE-O was examined along with well-being (WHO-5) and measures of symptom distress (SCL-10), and functioning (SDS-M).

**Results:**

Scores on all measures improved from pre- to post-treatment, except for functional impairment (SDS-M). Convergent validity was established with symptom distress (SCL-10; *r* = −0.63) and functioning (SDS-M; *r* = −0.55). A 10-point change in WHO-5 corresponded to an 18.9-point increase in Brief INSPIRE-O. ROC analysis identified an empirical cutoff of 50 for personal recovery and 8 points for clinically relevant change.

**Discussion:**

The Brief INSPIRE-O demonstrated strong validity and sensitivity to change, supporting its use as a reliable tool for assessing personal recovery and treatment quality in clinical practice. It can be considered a relevant brief patient reported outcome measure to be used in international standards of quality and outcome monitoring.

**Supplementary Information:**

The online version contains supplementary material available at 10.1007/s00127-025-02948-7.

## Introduction

The concept of personal recovery in mental health services has grown in importance in both research and clinical settings, encouraged by the World Health Organization [1, 2]. Personal recovery focuses on an individual’s journey towards a fulfilling life, beyond just managing mental health disorders. This concept requires services that respect each person’s unique experiences, helping them gain self-discovery and empowerment [3]. Although personal recovery is deeply individual, past studies have identified common elements such as connectedness, hope, identity, purpose, and empowerment as central to the recovery process [4, 5]. The challenge lies in distinguishing between “what characterizes personal recovery” and “factors that promote personal recovery,” as recovery is both a journey and a goal [6]. Consequently, measuring personal recovery remains difficult, as there are no universally recognized instruments for this purpose [7]. Whilst various tools exist, but none have emerged as a definitive benchmark [6, 7, 8, 9, 10, 11], particularly for non-psychotic disorders like depression and anxiety, where research is limited [6]. To integrate recovery-oriented practice with standard care, reliable and validated tools are needed to measure personal recovery [11, 12]. These tools should be simple enough for ongoing outcome measurements [13] and should meet international standards for quality monitoring [14]. One such tool is the 27-item user-rated INSPIRE measure, which has demonstrated reliability as a patient-rated experience tool for assessing recovery support [15, 16, 17]. A shorter version, the 5-item Brief INSPIRE, was adapted into a patient-reported outcome measure (PROM) called Brief INSPIRE-O [18] to assess personal recovery outcomes. The Brief INSPIRE-O has proven effective in measuring personal recovery, demonstrating its value as a reliable tool for assessing recovery in clinical settings [19]. However, there is tension between quantitatively assessing personal recovery and the individualized nature of the recovery process, which is often shaped by subjective experiences and goals. While it can be argued that quantifying recovery risks oversimplifying its complexity [22], empirical cutoffs and change scores on validated measures can provide valuable insights for clinical practice and research.

The study addresses the need for comprehensive validation of Brief INSPIRE-O across a broader mental health population, including non-psychotic disorders [6, 10]. It aligns with the COSMIN initiative’s standards for health measurement instruments, emphasizing the importance of capturing not only average treatment effects but also meaningful individual-level changes [20, 21].

The WHO-5, a widely used and validated measure of subjective well-being [22, 23], serves as a benchmark for establishing both cutoff scores and meaningful change thresholds, given its documented association with personal recovery [24]. In addition, convergent validity is assessed through the Hopkins Symptom Checklist (SCL-10) [25] and a modified version of the Sheehan Disability Scale (SDS-M) [26] capturing symptom distress and functional impairment, respectively.

To enhance clinical applicability, the study evaluates clinically significant and reliable change using the Reliable Change Index (RCI) and norm-based benchmarks derived from the general population. These methods offer a more precise understanding of whether observed individual changes exceed measurement error and reflect a transition toward normative recovery levels.

By integrating cutoff estimation, convergent validation, and reliable change analysis this study supports the Brief INSPIRE-O as a psychometrically sound tool for monitoring personal recovery. It offers clinicians and researchers a nuanced framework for evaluating treatment outcomes that reflect both individual improvement and broader recovery progress in mental health care.

## Method

### Study setting

Care Package (CP) treatments have been implemented for all nonpsychotic adult psychiatric outpatients in Denmark since 2010 (https://www.regioner.dk/sundhed/psykiatri-og-social/pakkeforloeb/). CP treatments are standardized, diagnosis-specific treatment courses with set resources [27, 28]. Mental Health Service, Capital Region of Denmark (MHS-CR) is the largest mental health service in Denmark, covering a catchment area of 1.85 million people with nine psychiatric treatment sites. To monitor treatment effects, MHS-CR has developed an internet-based monitoring system (IMS) collecting data pre- and post-treatment for all patients receiving an out-patient treatment for a non-psychotic disorder.

### Participants and procedure

The study used self-reported data collected through digital questionnaires sent to patients upon referral for treatment via the IMS. Psychiatrists and psychologists diagnosed patients as part of routine clinical practice, including those treated for depression, anxiety, and personality disorders. All data were recorded and stored following regional and national guidelines. The study included patients with complete IMS data from March 1, 2018, to March 1, 2020. Baseline data were used to categorize recovery levels, while pre- and post-treatment data were used for change score analysis. Fourteen days before treatment ended, patients received a link to complete post-treatment questionnaires, accessible for 30 days.

### Materials

The original INSPIRE measure assesses patient experiences of staff support for personal recovery based on the CHIME Framework, which identifies five recovery processes: Connectedness, Hope, Identity, Meaning, and Empowerment. The framework was validated by mental health service users and across cultures [15, 29, 30]. Both the Brief INSPIRE and full INSPIRE versions are internationally recognized and translated into 27 languages (www.researchintorecovery.com/inspire). We used the 5-item Brief INSPIRE-O, an adaptation of the Brief INSPIRE [31], as an outcome measure, changing references from professional support to personal outcomes. Scored on a 5-point Likert scale, it ranges from 0 (low recovery) to 100. The Danish version has shown reliable consistency in studies [18, 19].

Symptom distress was assessed using SCL-10 [25], comprised of 10 items from the comprehensive SCL-90-R [32]. The total scale scores of the SCL-10 are multiplied by 2.5 to range from 0 (low symptom load) to 100. Its accuracy in measuring symptom variations in patients receiving treatment for depression or anxiety has been verified [25].

We used a modified version of Sheehan’s Disability Scale [33] (SDS-M) to assess social functioning. This three-item measure evaluates disruptions in work, social, and family life, scoring from 0 (not at all) to 10 (extremely) for each area, with a total score range of 0 (low disability) to 30 (severe disability). The modification excludes reporting lost or unproductive days and adjusts wording to distinguish between broader social and intimate relationships. SDS-M is known for its strong psychometric properties and its ability to differentiate between active and inactive treatments, making it a reliable assessment tool [34].

The World Health Organization Well-Being Index (WHO-5) was used to assess subjective well-being. This self-assessment tool consists of 5 positive items rated from 0 (never) to 5 (all the time), with scores multiplied by 4, ranging from 0 (poor well-being) to 100 (optimal well-being). The WHO-5 has strong construct validity and reliably detects changes in subjective well-being across various settings [23]. We used two benchmarks: WHO-5 scores of ≥ 50 for good well-being [25] and ≥ 40 for acceptable well-being [18]. An increase of 10 points is considered a minimal clinical improvement, based on a systematic review [23].

### Statistical methods

Patient characteristics, including gender, age, and primary diagnosis, were presented for all patients with a Brief INSPIRE-O score at baseline (*n* = 8,192) and for those with scores at both baseline and follow-up (*n* = 2,714). ROC curves for Brief INSPIRE-O against WHO-5 ≥ 40 and WHO-5 ≥ 50 were produced for both genders, and areas under the curves (AUCs) were calculated. Optimal cutoff values for Brief INSPIRE-O were estimated using Youden’s and Liu’s criteria [35, 36]. Diagnostic performance was assessed via sensitivity, specificity, and overall agreement. Reliable change (RC) was estimated using the test-retest reliability of Brief INSPIRE-O from Moeller et al. [19], and the clinical significant change (CSC) value was estimated using Danish norm data of Brief INSPIRE-O based on Danish background population [18, 37]. The percentage of RCs exceeding 1.96 and percentage of follow-up Brief INSPIRE-O scores exceeding the CSC value were calculated for all patients with both baseline and follow-up scores, and for males and females separately. For patients with both baseline and follow-up scores, paired *t*-tests measured changes in Brief INSPIRE-O, SDS-M, SCL-10, and WHO-5. Convergent validity of Brief INSPIRE-O was analyzed using Pearson correlations with change scores of SDS-M, SCL-10, and WHO-5. Sensitivity to change in Brief INSPIRE-O was tested by comparing patients with an improvement on WHO-5 of at least 10 points [23] to those with less than 10 points improvement on the WHO-5 using independent t-tests, and Cohen’s d was calculated to evaluate effect size. Sensitivity analyses were conducted for all patients, each gender, and each diagnosis. ROC curves for Brief INSPIRE-O change scores against an improvement on the WHO-5 of ≥ 10 were produced, and AUCs were calculated. Optimal cutoff values were determined using Youden’s and Liu’s criteria, and diagnostic performance was assessed. All analyses were conducted using Stata version 18.0 [38], with a two-sided significance level of 5%.

## Results

Detailed demographic and diagnostic distributions are presented in Table 1. At baseline, 23.2% were male and 76.8% were female, which shifted to 22.2% male and 77.8% female among patients with data at both baseline and follow-up. The average age increased from 32.9 years (SD = 11.7) at baseline to 34.5 years (SD = 12.2) for those with both baseline and follow-up data. In terms of diagnosis distribution, at follow-up, there were perceptible increases in the percentages of patients with depression (from 33.7 to 38.0%) and anxiety (from 27.5 to 30.6%), in patients with data at both baseline and follow-up, while there was a decline in those diagnosed with a personality disorder (from 19.6 to 15.6%) and eating disorder (from 7.7 to 4.7%). The percentage of patients with PTSD remained constant at 9.6%. There were no significant differences between the group with both baseline and follow-up data and the group with only baseline data on any of self-report scales (see supplementary material).

**Table 1:** Patient characteristics at baseline

Characteristic	Patients included in analyses at	
	Baseline	Both baseline and follow-up
n	8192	2714
*Gender, n (%)*		
Male	1903 (23.2)	603 (22.2)
Female	6289 (76.8)	2111 (77.8)
Age, mean (SD)	32.9 (11.7)	34.5 (12.2)
*Diagnosis, n (%)*		
Depression	2556 (33.7)	782 (30.6)
Anxiety	2094 (27.5)	973 (38.0)
Personality disorder	1484 (19.6)	400 (15.6)
PTSD	725 (9.6)	246 (9.6)
Eating disorder	584 (7.7)	120 (4.7)
Other	142 (1.9)	38 (1.5)

Missing (n): diagnosis (baseline: 626; both baseline and follow-up: 155)

### Cutoff scores of Brief INSPIRE-O

**Fig. 1:**
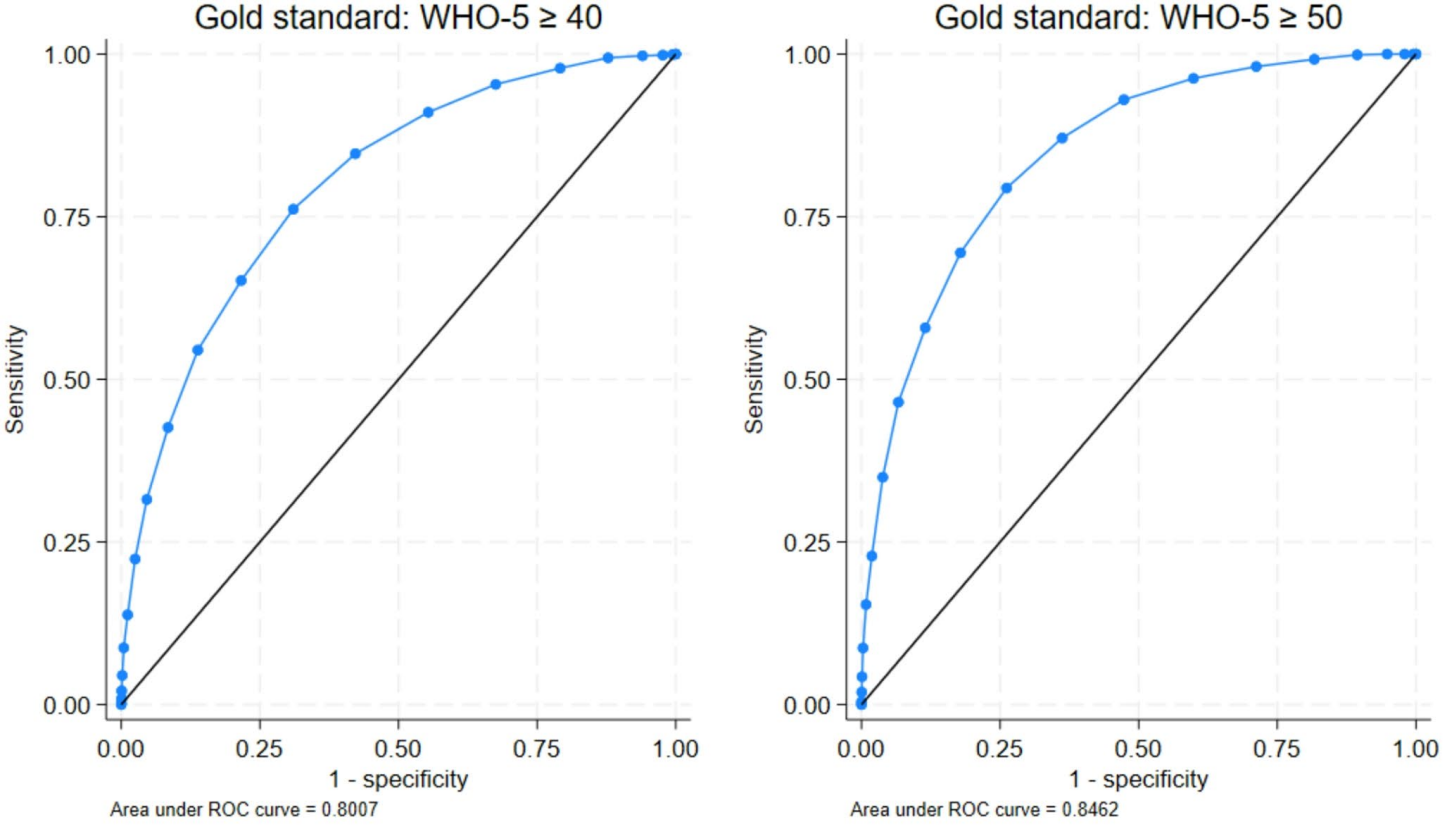
Receiver operating characteristic (ROC) curves of Brief Inspire-O against WHO-5 at baseline, *n* = 8192

Table 2; Fig. 1 present the diagnostic performance of the Brief INSPIRE-O scale using two WHO-5 benchmarks: WHO-5 ≥ 40 and WHO-5 ≥ 50. When using WHO-5 ≥ 40 as the gold standard, the Brief INSPIRE-O had an AUC of 80% (*n* = 8192), and the optimal cutoff was ≥ 45 (AUC = 72%, sensitivity = 76.0%, specificity = 68.9%, agreement = 70.6%). When using WHO-5 ≥ 50, the AUC increased to 85%, and with a cutoff of ≥ 50, the AUC was 76% (sensitivity = 79.2%, specificity = 73.8%, agreement = 74.4%). Cutoff values were identical for males and females, showing similar diagnostic performance. Sensitivity analyses confirmed the same cutoff values using Lui’s criterion.

**Table 2:** ROC-analyses of Brief INSPIRE-O scale on different benchmarks of WHO-5 at baseline, *n* = 8192

	Gold standard: WHO-5 ≥ 40					Gold standard: WHO-5 ≥ 50				
	Estimated cutoff value (≥)	Sensitivity in %	Specificity in %	Agreement in %	AUC (95%-CI)	Estimated cutoff value (≥)	Sensitivity in %	Specificity in %	Agreement in %	AUC (95%-CI)
All, *n* = 8192	45	76.0	68.9	70.6	0.72 (0.71, 0.74)	50	79.2	73.8	74.4	0.76 (0.75, 0.78)
*Stratified on gender*										
Males, *n* = 1903	45	75.1	69.2	70.5	0.72 (0.70, 0.74)	50	79.2	75.1	75.5	0.77 (0.74, 0.80)
Females, *n* = 6289	45	76.3	68.8	70.6	0.73 (0.71, 0.74)	50	79.2	73.4	74.1	0.76 (0.75, 0.78)

Abbreviation: AUC = Area under the curve, CI = confidence interval

Cutoff values estimated using Youden’s criterion

### Reliable and clinically significant change

As shown in Tables 3 and 24% of patients showed a Reliable Change (RC > 1.96) in Brief INSPIRE-O scores, indicating change beyond measurement error. Additionally, 38% of patients exceeded the Clinically Significant Change (CSC) threshold based on the Danish background population, suggesting a transition toward normative functioning. These proportions were consistent across genders, with slightly higher RC and CSC rates in males.

**Table 3:** Brief-INSPIRE-O reliable change values and clinically significant change values among patients with follow-up data, stratified on gender. (*n* = 2714)

	Baseline Mean (SD)	Follow up Mean (SD)	Reliable change^a^ *n* (%) of RC > 1.96	Clinically significant change^b^	
				Cutoff	*n* (%) > Cutoff
All	39.6 (17.1)	50.4 (21.0)	648 (23.9)	56.38	1017 (37.5)
*Stratified on gender*					
Males, *n* = 603	39.5 (16.7)	50.4 (21.8)	157 (26.0)	56.52	230 (38.1)
Females, *n* = 2111	39.6 (17.3)	50.3 (20.8)	491 (23.3)	56.29	787 (37.3)

Range of Brief INSPIRE-O (0-100)

^a^Using test-retest reliability of Brief INSPIRE-O: ICC = 0.75 [19]

^b^Using norm data of Brief INSPIRE-O based on Danish background population: mean (SD) = 71.1 (19.5) [18]

### Change scores from baseline to follow up

Significant improvements in Brief INSPIRE-O, SCL-10, SDS-M, and WHO-5 scores were observed from baseline to follow-up across all patients, as shown in Table 4. The Brief INSPIRE-O increased by an average of 10.8 points (95%-CI = (10.1, 11.5)), while WHO-5 increased by 16.1 points (95%-CI = (15.2, 16.9)). SDS-M showed a decrease in impaired functioning by −5.4 points (95%-CI = (−5.7, −5.1)), and SCL-10 demonstrated a reduction in symptom distress by −16.5 points (95%-CI = (−17.2, −15.7)). Similar patterns were observed across genders, though changes in SDS-M and SCL-10 were less favorable for personality disorder and PTSD. The largest improvement in Brief INSPIRE-O was seen in eating disorders (+ 15.1 points), while anxiety and PTSD showed the smallest increases. At follow-up, eating disorders had the highest mean score (61.7), while depression, personality disorder, and PTSD had the lowest scores (48.0, 46.9, 45.4, respectively).

### Convergent validity of Brief INSPIRE-O change scores

The correlations between changes in the Brief INSPIRE-O scores and changes in measures of symptoms (SCL-10), function (SDS-M), and subjective well-being (WHO-5) were all highly significant (*p* < 0.001) (Table 4). The Brief INSPIRE-O change score was negatively correlated with the SCL-10 change score (−0.63) and the SDS-M change score (−0.55). Conversely, the WHO-5 showed a positive correlation of 0.66 with the Brief INSPIRE-O. These patterns were consistent across gender and various diagnoses.

**Table 4:** Change in scales from baseline to follow-up among patients with follow-up data, stratified on gender and diagnosis. (*n* = 2714)

	Mean (SD)		Increase from baseline to FU	
	Baseline	Follow up	Difference Diff. (95%-CI)	Correlation with increase in Brief Inspire-O scale Correlation
**All**				
Brief Inspire-O, *n* = 2714	39.6 (17.1)	50.4 (21.0)	10.8 (10.1, 11.5)	1
SCL-10, *n* = 2709	58.1 (16.3)	41.6 (20.7)	−16.5 (−17.2, −15.7)	−0.63
SDS-M, *n* = 2709	20.6 (5.8)	15.2 (7.8)	−5.4 (−5.7, −5.1)	−0.55
WHO-5, *n* = 2711	26.5 (16.1)	42.5 (22.1)	16.1 (15.2, 16.9)	0.66
**Stratified on gender**				
*Males*				
Brief Inspire-O, *n* = 603	39.5 (16.7)	50.4 (21.8)	11.0 (9.5, 12.6)	1
SCL-10, *n* = 602	57.3 (16.1)	39.7 (20.6)	−17.6 (−19.2, −16.0)	−0.66
SDS-M, *n* = 602	20.6 (5.7)	15.0 (8.0)	−5.6 (−6.2, −5.0)	−0.60
WHO-5, *n* = 603	26.4 (16.0)	43.6 (22.7)	17.1 (15.3, 18.5)	0.71
*Females*				
Brief Inspire-O, *n* = 2111	39.6 (17.3)	50.3 (20.8)	10.7 (9.9, 11.5)	1
SCL-10, *n* = 2107	58.3 (16.4)	42.2 (20.8)	−16.1 (−17.0, −15.3)	−0.62
SDS-M, *n* = 2107	20.6 (5.8)	15.3 (7.7)	−5.3 (−5.7, −5.0)	−0.54
WHO-5, *n* = 2108	26.5 (16.2)	42.2 (22.0)	15.8 (14.8, 16.7)	0.65
**Stratified on diagnosis**				
*Depression*				
Brief Inspire-O, *n* = 973	35.0 (15.4)	48.0 (20.7)	13.1 (11.9, 14.2)	1
SCL-10, *n* = 973	58.7 (15.8)	40.2 (20.3)	−18.5 (−19.7, −17.3)	−0.64
SDS-M, *n* = 972	21.5 (5.5)	15.4 (7.7)	−6.1 (−6.6, −5.7)	−0.58
WHO-5, *n* = 973	21.1 (14.1)	41.7 (21.6)	20.6 (19.2, 22.0)	0.68
*Anxiety*				
Brief Inspire-O, *n* = 782	46.8 (16.9)	55.1 (19.9)	8.3 (7.1, 9.5)	1
SCL-10, *n* = 782	57.1 (16.2)	39.7 (19.5)	−17.4 (−18.7, −16.1)	−0.58
SDS-M, *n* = 782	19.7 (5.9)	14.1 (7.6)	−5.6 (−6.1, −5.0)	−0.50
WHO-5, *n* = 780	31.3 (16.5)	46.1 (21.1)	14.8 (13.4, 16.2)	0.60
*Personality disorder*				
Brief Inspire-O, *n* = 400	36.4 (16.2)	46.9 (20.7)	10.5 (8.6, 12.3)	1
SCL-10, *n* = 398	55.4 (16.3)	43.3 (20.3)	−12.2 (−14.1, −10.2)	−0.62
SDS-M, *n* = 398	20.4 (5.5)	15.4 (7.7)	−4.3 (−5.0, −3.6)	−0.59
WHO-5, *n* = 399	28.6 (15.8)	41.7 (21.6)	11.8 (9.7, 13.9)	0.64
*PTSD*				
Brief Inspire-O, *n* = 246	37.0 (16.5)	45.4 (22.3)	8.4 (5.7, 11.1)	1
SCL-10, *n* = 244	62.4 (16.5)	50.1 (22.9)	−12.1 (−14.9, −9.4)	−0.66
SDS-M, *n* = 245	22.0 (5.8)	18.2 (7.9)	−3.8 (−4.9, −2.7)	−0.57
WHO-5, *n* = 246	22.9 (17.8)	34.1 (23.8)	11.3 (8.2, 14.3)	0.69
*Eating disorder*				
Brief Inspire-O, *n* = 120	46.6 (16.7)	61.7 (20.1)	15.1 (11.7, 18.6)	1
SCL-10, *n* = 120	59.4 (16.5)	37.4 (22.3)	−21.9 (−26.3, −62.3)	−0.68
SDS-M, *n* = 120	18.3 (6.4)	11.5 (7.9)	−6.8 (-8.3, −5.2)	−0.54
WHO-5, *n* = 120	35.8 (15.5)	53.8 (22.5)	18.0 (8.2, 14.3)	0.66

Range of scales: Brief Inspire-O (0-100), SCL-10 (0-100), SDS-M (0–30), WHO-5 (0-100)

All correlations are highly statistically significant (*p* < 0.001)

### Sensitivity to change

Table 5 displays the sensitivity of the Brief INSPIRE-O scale to detect changes in clinical improvement in the WHO-5. For the entire sample (*n* = 2714), those with a WHO-5 improvement of ≥ 10 points saw a significant increase of 18.9 points (95%-CI = (18.1, 19.8)) in Brief INSPIRE-O, compared to a slight decrease of −0.3 points in those with less improvement. The overall difference between these groups was 19.18 points (95%-CI = (17.96, 20.40)), with a large effect size (Cohen’s *d* = 1.20). Males (*n* = 603) showed a 20.4-point increase (95%-CI = (18.6, 22.3)) in the higher WHO-5 improvement group, while females (*n* = 2111) showed an 18.5-point increase (95%-CI = (17.5, 19.4)). Diagnosis-specific analyses showed the largest effect size for PTSD (1.36) and the smallest for anxiety (0.99).

**Table 5:** Sensitivity to change: change in brief Inspire-O scale according to clinical improvement in WHO-5 of at least 10 scale points. (*n* = 2714)

	WHO-5 improvement < 10, *n* = 1155			WHO-5 improvement ≥ 10, *n* = 1556			Difference in increases, *n* = 2714	
	Brief Inspire-O at baseline Mean (SD)	Brief Inspire-O at follow-up Mean (SD)	Increase from baseline to follow-up Mean (95%-CI)	Brief Inspire-O at baseline Mean (SD)	Brief Inspire-O at follow-up Mean (SD)	Increase from baseline to follow-up Mean (95%-CI)		
							Mean (95%-CI)	Cohen’s d^a^
All, *n* = 2714	39.6 (17.7)	39.4 (18.4)	−0.3 (−1.1, 0.5)	39.6 (16.7)	58.5 (19.0)	18.9 (18.1, 19.8)	19.18 (17.96, 20.40)	1.20
*Stratified on gender*								
Male, *n* = 603	40.1 (17.2)	38.6 (18.7)	−1.6 (−3.3, 0.2)	39.0 (16.3)	59.5 (19.8)	20.4 (18.6, 22.3)	21.99 (19.33, 24.64)	1.34
Female, *n* = 2111	39.5 (17.9)	39.6 (18.3)	0.1 (−0.8, 1.0)	39.7 (16.8)	58.2 (18.9)	18.5 (17.5, 19.4)	18.37 (17.00, 19.75)	1.16
*Stratified on package/diagnosis*								
Anxiety, *n* = 782	46.5 (17.8)	46.4 (19.2)	−0.2 (−1.6, 1.2)	47.0 (16.3)	61.7 (17.8)	14.7 (13.2, 16.3)	14.89 (12.76, 17.02)	0.99
Depression, *n* = 973	34.3 (15.6)	34.9 (16.9)	0.6 (−0.7, 2.0)	35.3 (15.3)	55.4 (19.0)	20.1 (18.7, 21.4)	19.45 (17.38, 21.53)	1.23
Personality disorder, *n* = 400	37.5 (17.0)	37.8 (16.6)	0.3 (−1.8, 2.3)	35.4 (15.4)	55.6 (18.4)	20.2 (17.8, 22.6)	19.92 (16.72, 23.11)	1.23
Eating disorder, *n* = 120	46.7 (15.2)	48.4 (15.9)	1.7 (−2.8, 6.2)	46.5 (17.6)	69.4 (18.2)	22.9 (18.9, 26.8)	21.19 (15.07, 27.31)	1.30
PTSD, *n* = 246	36.4 (17.2)	33.2 (16.8)	−3.2 (−6.0, −0.4)	37.6 (15.8)	58.8 (19.8)	21.1 (17.5, 24.7)	24.29 (19.81, 28.77)	1.36

^a^As a general rule of thumb, *d* = 0.2 may be regarded as small, *d* = 0.5 as medium, and *d* = 0.8 as large

### Cutoff for Brief INSPIRE-O change score

Using the minimal clinically important difference in WHO-5 of 10 points or more as an indicator of relevant change [23], Table 6 shows the corresponding performance of the Brief INSPIRE-O. For all participants, the AUC of the Brief INSPIRE-O change score against WHO-5 increase ≥ 10 was 0.73, and the optimal cutoff value of the Brief INSPIRE-O change score was 8, with sensitivity, specificity, and agreement percentages around 73%. When stratified by gender, males show slightly higher sensitivity, specificity, and agreement, as well as a higher AUC (0.76), compared to females (AUC of 0.72).

**Table 6:** ROC-analyses of change in brief Inspire-O against clinical improvement in WHO-5 of at least 10 scale points. (*n* = 2714)

	Gold standard: Improvement in WHO-5 ≥ 10				
	Estimated cutoff value (≥)	Sensitivity in %	Specificity in %	Agreement in %	AUC (95%-CI)
All, *n* = 2714	8	73.8	72.1	73.1	0.73 (0.71, 0.75)
*Stratified on gender*					
Males, *n* = 603	8	77.4	75.6	76.6	0.76 (0.73, 0.80)
Females, *n* = 2111	8	72.8	71.1	72.1	0.72 (0.70, 0.74)

Abbreviation: AUC = Area under the curve, CI = confidence interval

Cutoff values estimated using Youden’s criterion

## Discussion

This study assessed the Brief INSPIRE-O as a measure of personal recovery in a non-psychotic mental health population, demonstrating its sensitivity to change. Of the 8,192 patients at baseline, 2,714 had data at both baseline and follow-up, a retention rate consistent with similar studies [39].

### Cutoff for Brief INSPIRE-O change score

Determining clinical cutoff values is an important aspect of measurement tools. This study explored cutoff values for the Brief INSPIRE-O scale using two benchmarks of the WHO-5 scale: ≥ 40 and ≥ 50. The WHO-5 benchmark of ≥ 50, derived from a macroanalysis of a mixed clinical sample, categorized treatment needs into “None or Mild (≥ 50),” “Moderate (49–26),” and “Severe (< 26)” [25]. Additionally, the WHO-5 benchmark of ≥ 40 falls within a normal range [18], guiding the use of both benchmarks for determining cutoff values.

The study found that a Brief INSPIRE-O score of ≥ 45 had good sensitivity and specificity with the WHO-5 ≥ 40 benchmark, but performance improved when using the WHO-5 ≥ 50 benchmark. A Brief INSPIRE-O cutoff of ≥ 50 demonstrated better diagnostic accuracy, making it a recommended indicator of “good” personal recovery.

It is important to remember that clinical cutoffs provide only a snapshot of recovery, and individuals may move in and out of recovery over time. Sustained personal recovery, rather than a single score, is more reflective of long-term outcomes like quality of life and well-being, emphasizing recovery as a continuous journey.

### Change scores and convergent validity

This study found significant improvement in Brief INSPIRE-O scores from baseline to follow-up, with individuals diagnosed with eating disorders showing the greatest improvement. Anxiety and PTSD showed more modest gains, while depression, personality disorders, and PTSD had the lowest baseline scores but also improved by the end of treatment. Both WHO-5 and Brief INSPIRE-O scores reached normative ranges post-treatment [18], affirming their utility in monitoring mental health interventions. However, SDS-M scores showed persistent functional impairment compared to Denmark’s normative levels [18, 40], and SCL-10 scores decreased significantly, indicating overall clinical improvement [18]. A key finding is the strong negative correlation (*r* = −0.63) between changes in personal recovery (Brief INSPIRE-O) and symptom distress (SCL-10), suggesting that reductions in distress are linked to improvements in personal recovery. This contrasts with previous research in psychotic disorders, which reported weaker correlations between levels of personal recovery and symptom distress [41]. Our study’s focus on change scores offers a fresh perspective, indicating that improvements in symptom distress are closely related to changes in personal recovery, aligning with recent reviews on recovery from eating disorders that emphasize the importance of integrating clinical improvement with personal recovery [42]. A strong negative correlation (*r* = −0.55) was also found between changes in functional impairment (SDS-M) and personal recovery, which differs from findings in psychotic disorders where functional recovery was more easily achieved than personal recovery [41]. This suggests that for non-psychotic patients, personal recovery might be more accessible and could serve as a foundation for later functional recovery.

Our findings indicate that in non-psychotic populations, personal recovery may be prioritized before functional recovery. This observation is supported by the strong association (*r* = 0.66) between increases in WHO-5 and Brief INSPIRE-O, highlighting that improvements in subjective well-being align with personal recovery across diagnoses and genders.

### Reliable and clinically significant change

Our findings demonstrate that the Brief INSPIRE-O can detect meaningful individual-level improvements in personal recovery. Approximately one in four patients showed reliable change, indicating that their improvement exceeded the threshold for measurement error. Reliable change refers to statistical confidence that change has occurred beyond what might be expected from measurement variability alone. In addition, over one-third of patients had an improvement of personal recovery scores above the cutoff for clinically significant change, based on normative data from the general Danish population [18]. While this does not imply that patients reached the normative mean (a score of 71), it reflects a substantial and meaningful shift toward typical recovery levels. Clinically significant change refers to an improvement that not only is statistically reliable, but also crosses a threshold based on normative population data indicating a shift from a clinical to a more typical or non-clinical level of functioning.

Combining ROC-derived thresholds with the Clinically Significant and Reliable Change (CSRC) approach offers a more robust framework for interpreting change in personal recovery. While ROC thresholds provide absolute cutpoints that support generalizable interpretation across settings, the CSRC approach is sample-specific and does not extend beyond the current study. These distinct functions underline the value of using both methods to balance individual-level insight with broader clinical applicability.

### Sensitivity to change and cutoff for Brief INSPIRE-O change score

This study highlights the Brief INSPIRE-O scale’s sensitivity to change over time, demonstrating a significant increase in scores among patients with improved well-being, as measured by the WHO-5 scale. The large effect size (Cohen’s *d* = 1.20) supports its utility in detecting clinically meaningful improvements. Based on the WHO-5 criterion of a 10-point improvement, a Brief INSPIRE-O change score of 8 is recommended as a meaningful cutpoint for both genders. While effect sizes varied slightly across diagnostic groups, ranging from *d* = 0.99 (anxiety) to *d* = 1.36 (PTSD), the scale consistently demonstrated sensitivity to change across all groups. This suggests that the Brief INSPIRE-O is a robust and broadly applicable measure for tracking personal recovery in diverse nonpsychotic clinical populations.

### Clinical implications

The Brief INSPIRE-O scale can serve as a valuable tool for monitoring mental health changes in clinical settings that prioritize a holistic care, emphasizing the patient´s rights to personal growth and ”lives worth living” from the patient perspective. Its sensitivity to change makes it a promising instrument for assessing the impact of interventions targeting personal recovery. Additionally, the determination of clinical cutoff values provides a practical guide for clinicians to interpret Brief INSPIRE-O scores and changes in scores during treatment. By identifying both reliable and clinically significant change at the individual level, the scale enables practitioners to evaluate whether a patient has meaningfully progressed in their recovery journey beyond group averages thus supporting more personalized and responsive care.

### Implications for mental health services

One potential contribution of this study is to contribute to enhancing holistic and person-centered care in mental health services by offering validated measures of personal recovery. By emphasizing personal recovery, this study could help balance the focus between managing clinical symptoms and addressing the broader aspects of patients’ well-being in mental health services. However, any effort to quantify recovery requires caution in its use, in order to maintain the person-centred value which is central to a recovery orientation [43]. By introducing a validated measure of personal recovery, our study may influence how mental health interventions are evaluated, supporting a shift from solely treating disorder to promoting well-being and quality of life. This aligns with a broader societal view that values personal development, fulfilment, and meaningful participation in life.

### Strengths, limitations, and future research

This study, using a large and diverse sample from Denmark’s largest mental health hospital over two years, provides a comprehensive view of the Brief INSPIRE-O’s utility across various mental health conditions. A key strength is the practical guideline offered for clinicians, directly applicable in everyday practice. Despite the challenges often associated with “real-world data,” this dataset is relatively homogeneous, thanks to consistent data collection methods across identified patient categories. However, the high level of missing data at the post-treatment time point poses a limitation, potentially biasing results and excluding certain patient groups, which could affect the accuracy of change scores.

While the Brief INSPIRE-O offers valuable quantitative insights at the team and service levels, it cannot fully capture individual subjective experiences. A blend of quantitative and qualitative methods, such as interviews and patient diaries, would provide a more complete understanding of personal recovery. The absence of patients with psychotic experiences in the sample highlights the need for further research to assess the scale’s applicability in such populations.

Future research should investigate potential differences across diagnoses, compare the Brief INSPIRE-O with other validated recovery measures, and explore the relationship between clinical, personal, and functional recovery over time. Repeated measurements in follow-up studies could provide deeper insights into how personal recovery interact with clinical and functional recovery in various patient populations, enhancing the understanding and application of personal recovery in mental health care.

### Conclusion

In conclusion, this study validates the Brief INSPIRE-O scale’s sensitivity to change and clinical utility. A score of 50 or more indicates personal recovery, while a change score of 8 suggests clinically relevant improvement. The Brief INSPIRE-O shows promise as a brief PROM for assessing treatment quality and could be used in international outcome monitoring standards [13, 14].

## Electronic supplementary material

Below is the link to the electronic supplementary material.

Supplementary Material 1

## Data Availability

Available through contact to corresponding author.
