# Supplementary material for: Assessing change and establishing empirical cutoffs: the Brief INSPIRE-O measure for personal recovery in mental health services
Source: Soc Psychiatry Psychiatr Epidemiol. 2025 Jun 24;60(11):2707–17. doi: 10.1007/s00127-025-02948-7 (PMC12572006; doi:10.1007/s00127-025-02948-7)
Supplement: Supplementary file 1 — Supplementary Material 1 [file 127_2025_2948_MOESM1_ESM.docx]

## Supplementary material

## Supplementary Table 1 Characteristics of participants included in baseline-analyses only and of participants included in all analyses

| **Characteristic** | **Included only in analyses**  **at baseline** | **Included in all analyses** | **Test of difference,**  ***p*-value** |
| --- | --- | --- | --- |
| n | 5478 | 2714 |  |
| **Gender, n (%)** |  |  | 0.127 |
| Male | 1300 (23.7) | 603 (22.2) |  |
| Female | 4178 (76.3) | 2111 (77.8) |  |
| **Age, mean (SD)** | 32.1 (11.4) | 34.5 (12.2) | <0.001 |
| **Diagnosis, n (%)** |  |  | <0.001 |
| Depression | 1583 (31.5) | 782 (30.6) |  |
| Anxiety | 1312 (26.1) | 973 (38.0) |  |
| Personality disorder | 1084 (21.6) | 400 (15.6) |  |
| PTSD | 479 (9.5) | 246 (9.6) |  |
| Eating disorder | 464 (9.2) | 120 (4.7) |  |
| Other | 104 (2.1) | 38 (1.5) |  |
| **Self-report scales, mean (SD)** |  |  |  |
| Brief Inspire-O | 40.0 (17.9) | 39.6 (17.1) | 0.262 |
| SCL-10 | 57.7 (17.3) | 58.1 (16.3) | 0.377 |
| SDS | 20.2 (6.2) | 20.6 (5.8) | 0.024 |
| WHO-5 | 27.5 (17.0) | 26.5 (16.1) | 0.024 |

Missing (n): diagnosis (baseline only: 452; both baseline and follow-up: 155), SCL-10 (baseline only: 16, both baseline and follow-up: 5), SDS (baseline only: 26, both baseline and follow-up: 5), WHO-5 (baseline only: 3, both baseline and follow-up: 3)
